# Detailed transcriptome analysis of the plant growth promoting *Paenibacillus riograndensis* SBR5 by using RNA-seq technology

**DOI:** 10.1186/s12864-017-4235-z

**Published:** 2017-11-03

**Authors:** Luciana Fernandes Brito, Marta Irla, Jörn Kalinowski, Volker F. Wendisch

**Affiliations:** 1 Department of Genetics of Prokaryotes, Faculty of Biology, Bielefeld University, Universitätsstraße 25, 33615 Bielefeld, Germany; 2 Department of Biotechnology and Food Science, Norwegian University of Science and Technology, Trondheim, Norway; 3 Center for Biotechnology (CeBiTec), Bielefeld University, Bielefeld, Germany

**Keywords:** *Paenibacillus riograndensis*, RNA sequencing, Transcriptional start sites, Promoter motifs, Ribosome biding sites, Operon structures, Thiamine pyrophosphate riboswitch *Paenibacillus sonchi*

## Abstract

**Background:**

The plant growth promoting rhizobacterium *Paenibacillus riograndensis* SBR5 is a promising candidate to serve as crop inoculant. Despite its potential in providing environmental and economic benefits, the species *P. riograndensis* is poorly characterized. Here, we performed for the first time a detailed transcriptome analysis of *P. riograndensis* SBR5 using RNA-seq technology.

**Results:**

RNA was isolated from *P. riograndensis* SBR5 cultivated under 15 different growth conditions and combined together in order to analyze an RNA pool representing a large set of expressed genes. The resultant total RNA was used to generate 2 different libraries, one enriched in 5′-ends of the primary transcripts and the other representing the whole transcriptome. Both libraries were sequenced and analyzed to identify the conserved sequences of ribosome biding sites and translation start motifs, and to elucidate operon structures present in the transcriptome of *P. riograndensis*. Sequence analysis of the library enriched in 5′-ends of the primary transcripts was used to identify 1082 transcription start sites (TSS) belonging to novel transcripts and allowed us to determine a promoter consensus sequence and regulatory sequences in 5′ untranslated regions including riboswitches. A putative thiamine pyrophosphate dependent riboswitch upstream of the thiamine biosynthesis gene *thiC* was characterized by translational fusion to a fluorescent reporter gene and shown to function in *P. riograndensis* SBR5.

**Conclusions:**

Our RNA-seq analysis provides insight into the *P. riograndensis* SBR5 transcriptome at the systems level and will be a valuable basis for differential RNA-seq analysis of this bacterium.

**Electronic supplementary material:**

The online version of this article (10.1186/s12864-017-4235-z) contains supplementary material, which is available to authorized users.

## Background

Members of *Paenibacillus* genus are Gram-positive, spore-forming, motile and facultatively anaerobic bacteria [1]. This group is biochemically and morphologically diverse and is found in various environments, such as soil [2], rhizosphere [3], insect larvae [4], and clinical samples [5]. Originally, *Paenibacillus* belonged to the genus *Bacillus*, however, in 1993 it was reclassified as a separate genus [6]. The important plant growth promoting (PGP) species *P. polymyxa*, *P. macerans* and *P. azotofixans* were included in the new genus when it was proposed [6]. The genus *Paenibacillus* currently comprises more than 150 named species; approximately 6% of these are able to fix nitrogen and possess some other plant growth promotion abilities [7].

*Paenibacillus riograndensis* SBR5 is the type strain of this species and was isolated from rhizosphere of wheat (*Triticum aestivum*) fields in the south of Brazil (Rio Grande do Sul) [8]. It was shown that *P. riograndensis* SBR5 is a promising candidate for crop inoculation because of its nitrogen fixation ability and other plant growth promotion characteristics such as production of phytohormones and antimicrobial substances [9, 10]. Furthermore, SBR5 is cellulolytic and xylanolytic, and is able to perform competition against Gram-negative and Gram-positive pathogens such as *Pectobacterium carotovorum* and *Listeria monocytogenes*, respectively [11].

A phylogenetic analysis of SBR5 based on the 16S rRNA gene sequence has showed that it is most closely related to *Paenibacillus graminis* RSA19^T^ (98.1% similarity) [9]. The genome of SBR5 was completely sequenced and annotated; its circular chromosome consists of 7,893,056 base pairs, with GC content of 50.97% [12]. The annotation of the finished genome sequence showed the presence of 6705 protein coding genes, 87 tRNAs and 27 rRNAs genes [12].

Recent research efforts on transcriptome characterization in paenibacilli focused on comparative transcriptomic analysis under different plant-related conditions [13, 14]. Contrary to these differential transcriptomic analyses, comprehensive transcriptome analysis allows to chart the RNA landscape of a particular organism for improvement of the genome annotation, detection of novel transcripts and conserved sequence motifs such as transcription start sites (TSS), promoters and ribosomal biding sites (RBS) [15, 16]. These comprehensive analyses have been performed for bacteria of industrial or public health relevance such as *Corynebacterium glutamicum* [15] and *Salmonella* [17]. Although complete genome sequences of several *Paenibacillus* PGP members have been published [12, 18–20], a genome-wide analysis of the transcriptome characterizing the whole transcriptome of a member of *Paenibacillus* genus is still missing.

In this study, we describe genome-wide TSS mapping and whole transcriptome analysis of *P. riograndensis* SBR5 cultivated under 15 conditions. Conserved sequence motifs for promoters, ribosome binding sites, riboswitches and other RNA families were determined, and the function of a TPP (thiamine pyrophosphate) riboswitch confirmed by translational fusion with a green fluorescence protein (GfpUV) reporter gene. TPP riboswitches typically bind TPP and regulate expression of genes that are involved in biosynthesis and transport of thiamine in eukaryotes and bacteria making them interesting targets for the study of antibacterial compounds [21].

## Methods

### Cultivation of *P. riograndensis* SBR5 in different conditions

*P. riograndensis* SBR5, the bacterial strain used in this study, was obtained from the strain collection of the Department of Genetics at Universidade Federeal do Rio Grande do Sul. Here, we exposed SBR5 to varied growth conditions. In all experiments, the bacterial cells were grown in 500 mL flasks containing 50 mL of medium shaking at 120 rpm and at 30 °C, if not stated otherwise. For each condition tested, four biological replicates were used: one for harvesting of bacterial cells and total RNA isolation, and three for further determination of growth characteristics. The optical density at 600 nm (OD_600 nm_) of the cultivated cells was measured throughout growth. The initial OD_600 nm_ in all cultivations was approximately 0.05.

The first experiment was performed with lysogeny broth (LB) as growth medium; the cells were grown under 3 different temperatures: 20 °C, 30 °C or 37 °C. Cells were also cultivated at 30 °C for further application of 5 min-cold shock (from 30 °C to 4 °C) or heat shock (from 30 °C to 50 °C) when the middle of the exponential phase was reached. The PbMM (*P. riograndensis* minimal medium) with 20 mM glucose as carbon source was used for application of the remaining stress conditions. Minimal PbMM medium contained the following, in 1 L of RO-water: K_2_HPO_4_, 4.09 g; NaH_2_PO_4_, 1.3 g; (NH_4_)_2_SO_4_, 2.11 g; biotin, 0.1 mg; concentrated trace element (TE) solution, 1 mL. The concentrated TE solution contained the following, in 1 L of RO-water: FeSO_4_*7H_2_O, 5.56 g; CuCl_2_*2H_2_O, 0.027 g; CaCl_2_*2H_2_O, 7.35 g; CoCl_2_*6H_2_O, 0.04 g; MnCl_2_*4H_2_O, 9.90 g; ZnSO_4_*7H_2_O, 0.288 g; Na_2_MoO_4_*2H_2_O, 0.048 g; H_3_BO_3_, 0.031 g. The growth of SBR5 was carried with addition of 100 mM KCl or NaCl or addition of 2 g L^−1^ of ethanol or methanol to the medium. Moreover, growth in PbMM with addition of 3 different carbon sources of was compared: 20 mM of glucose, 40 mM of glycerol or 10 mM of sucrose. Finally, the cells were cultivated in 3 different pHs: 5, 7 or 8, buffered with 50 mM of 2-(N-morpholino)ethanesulfonic acid (MES), 3-morpholinopropane-1-sulfonic acid (MOPS) and 3-[[1,3-dihydroxy-2-(hydroxymethyl)propan-2-yl]amino]propane-1-sulfonic acid (TAPS), respectively.

The bacterial cells were harvested in the middle of the exponential phase (Additional file 2: Table S2) and the harvesting procedure was done according to Irla et al. 2015 [16].

For the cultivation of the *P. riograndensis* transformants harboring plasmid DNA with *gfpUV* reporter gene under control of the pyruvate kinase promoter (Ppyk) with either native 5′ UTR (pP2pyk-*gfpUV*) or 5' untranslated regions (5′ UTR) of the gene P.riograndensis_final_150 (pP2pyk_TPP-*gfpUV*), the cells were routinely grown at 30 °C, shaking at 120 rpm, in medium DSMZ 220 [22] with addition of 5.5 μg mL^−1^ of chloramphenicol. *Escherichia coli* strains were routinely cultivated at 37 °C in LB supplied with 15 μg mL^−1^ of chloramphenicol when needed. To assay the effect of thiamine on *gfpUV* expression by the 2 *P. riograndensis* strains, bacterial cells were transferred from DSMZ 220 medium to glucose minimal medium PbMM (see above) with 0, 5, 10, 15, 20 or 25 μM of thiamine for SBR5(pP2pyk_TPP-*gfpUV*) and 0 or 25 μM of thiamine for SBR5(pP2pyk-*gfpUV*). After overnight growth, cells in minimal medium were used to inoculate fresh PbMM medium containing its respective thiamine concentration.

### RNA isolation and preparation of cDNA libraries for sequencing

In order to isolate total RNA from SBR5 cells, bacterial cell pellets previously harvested and kept at −80 °C were thawed in ice and RNA was extracted individually for each cultivation condition using NucleoSpin RNA isolation kit (Macherey-Nagel, Düren, Germany). Polymerase Chain Reactions (PCRs) utilizing Taq polymerase (New England Biolabs) and 2 pairs of primers amplifying 2 different genome regions was perform to detect the presence of remaining genomic DNA in the samples. Primer characteristics and sequences are listed in Additional file 1: Table S1 and the reactions were carried according to the Taq polymerase manufacture’s recommendations. RNA samples with genomic DNA contamination were treated with the RNase-free DNase set (Qiagen, Hilden, Germany). The concentration of isolated RNA was determined by DropSense™ 16 (Trinean, Ghent, Belgium; software version 2.1.0.18). To verify the quality of RNA samples, we performed capillary gel electrophoresis (Agilent Bioanalyzer 2100 system using the Agilent RNA 6000 Pico kit; Agilent Technologies, Böblingen, Germany). All procedures to obtain high quality RNA were done according to manufacturer’s recommendations. The extracted RNA samples were pooled in equal parts and the pool of total RNA was subsequently used for the preparation of 2 different cDNA libraries.

The cDNA libraries of SBR5 were prepared according to 2 different protocols. One library followed the protocol for the enrichment of 5′-ends of primary transcripts, while the other method allowed the analysis of the whole transcriptome [15, 16]. The libraries were prepared and sequenced according to Irla et al. 2015 [16]. The generated whole transcriptome and 5′-end enriched cDNA libraries were sequenced on a single flow cell of a MiSeq Desktop Sequencer system.

### Mapping sequenced reads onto the genome of *P. riograndensis* SBR5

Before mapping to the reference genome, the reads obtained during sequencing of the whole transcriptome and 5′-end enriched library were trimmed to a minimal length of 20 base pairs with the Trimmotatic ver. 0.33 [23], with three first base pairs cut off at the start and bad quality bases at the end of the reads. The reads of 5′-end enriched library were trimmed in the single end mode, whereas those of whole transcriptome library in paired end mode. Trimmed reads were mapped to the reference genome of *P. riograndensis* SBR5 (accession number LN831776.1) by using the software for short read alignment Bowtie [24].

### Determination of transcription start sites (TSS) based on 5′-end enriched library

To determine and classify the TSS based on mapped 5′-end enriched RNA-seq data, we used the software for visualization of mapped sequences ReadXplorer [25]. This determination was done in 2 steps, automatic TSS determination and manual data set curing. First, the TSS were automatically detected by ReadXplorer Transcription Analysis Parameter Wizard, following 2 different selected sets of criteria described in Table 1. In the generated data, to each TSS detected, several characteristics were reported; including: 70 base pairs sequence upstream the TSS, the assigned gene name and product, the DNA strand to which the assigned gene belongs, the assigned gene start and end position, the distance between the given TSS and its assigned translation start sites (TLS) and its classification regarding a TSS assigned to tRNA, mRNA or a novel transcript. As second step, the data generated through the 2 parameter sets were combined and manually cross-checked to classify the novel transcripts as antisense, intergenic or intragenic, and also to eliminate false positives, as previously described by Irla et al. 2015 [16].

**Table 1:** Parameter sets selected for transcription analysis of *P. riograndensis* SBR5

Transcription start site detection parameters	1	2
Minimum number of read starts	5	3
Minimum percent of coverage increase	48	48
Maximum low coverage read start count	0	20
Minimum low coverage read starts	0	3
Minimum transcript extension coverage	20	5
Maximum distance to feature of leaderless transcripts	300	5500
Associate neighboring TSS in a base pair window of	3	3

### Determination of 5′ UTR length and identification of cis-regulatory elements in 5′ UTRs of *P. riograndensis* SBR5 genes

A genome-wide analysis was performed in order to identify putative RNA motifs in the genome of SBR5. To this end, we used the Infernal tool [26]. The RNAs were annotated to the genome of SBR5 in conjunction with the Rfam database [27]. Furthermore, based on the difference between the position of the analyzed TSS and its assigned TLS, we could determine the 5′ UTR length of each TSS belonging to an annotated gene. The 5′ UTRs which were longer than 100 base pairs were used as candidates to evaluate whether they contain *cis*-regulatory elements. In total, 209 5′ UTRs were analyzed by comparison to Rfam database [28]. Because thiamine is involved in the interaction of plants with plant growth promoting rhizobacteria [29], a TPP riboswitch was selected among the detected riboswitches for further analysis; a 313 base pairs sequence of the TPP riboswitch present in the 5′ UTR of the *thiC* gene was analyzed in the ARNold tool for identification of transcriptional terminators [30] and in the RNAfold tool for determination of its secondary structure [31].

### Detection of conserved ribosomal biding site (RBS) and promoter motifs sequences

To identify the conserved promoter motifs, 70 base pairs sequences upstream the TSS assigned to annotated genes were analyzed. All the genes with identified TSS were considered in the analysis of TLS and RBS motifs, for this analysis 50 base pairs upstream of TLS were considered. The Improbizer [32] program was used to find the motifs and the tool WebLogo [33] was used to generate the visualization charts. In both programs, the default settings were applied for the analysis. In the text representations, the conserved motifs are represented in upper or lower case depending on its conservation, as follows: nucleotides in upper case letters represent more than 80% of occurrence among all analyzed sequences, nucleotides in lower case letters represent occurrence of more than 40%, but less than 80% of all cases. If a base occurs less often than 40%, the letter “n” in lower case appears.

### Determination of most abundant genes transcribed in *P. riograndensis* SBR5

In order to determine the most abundant genes transcribed in the applied cultivation conditions in SBR5, the whole transcriptome RNA-seq data set was used. The data was normalized by calculation of Reads Per Kilobase per Million mapped reads (RPKM) [34]. The calculation of abundances was automatically generated by the ReadXplorer software [25] as described in Irla et al. 2015 [16]. When the transcripts of proteins of unknown function were automatically defined as the most abundant, the gene sequences were submitted to BLASTx analysis to identify the family to which the protein in question belongs [35].

### Identification of operon structures in *P. riograndensis* SBR5

The operon structures present in this transcript analysis were automatically detected in the ReadXplorer software [25]. The same approach was previously shown in Irla et al. 2015 [16]. The classical operon has multiple genes transcribed as a single mRNA molecule having a single promoter to drive its expression, but transcription start sites internal to the operon sequence pointed to the presence of suboperons which often respond to different conditions [15, 16]. Based on the whole transcriptome RNA-seq data set, an operon structure was identified if the intergenic space of 2 genes positioned in same orientation linked those genes by a bridge of at least 2 paired mappings. Among the detected operon structures, the operons and suboperons were classified separately: a primary operon was considered when a TSS was assigned to the first gene of the operon; and a suboperon was detected when a TSS was assigned within primary operons. Furthermore, the automatically generated operon set was manually cross-checked with the complete whole transcriptome RNA-seq data. Finally, the difference between the position in the genome of the first nucleotide and the last nucleotide of the suboperons/operons was calculated to determine the approximated suboperons/operons length distribution. This calculation does not take the lengths of 5′ UTRs and 3′ UTRs into account.

### Strains, plasmid construction and primers

*P. riograndensis* SBR5 was used as host for heterologous expression of *gfpUV*. Information about the plasmids constructed in this work and primer sequences is available in Additional file 1: Table S1. Molecular cloning was performed as described by Sambrook (2001) [36]. Chemically competent cells of *E. coli* DH5α were prepared for cloning [37]. Genomic DNA of *P. riograndensis* SBR5 was isolated as described by Eikmanns et al. (1994) [38]. The NucleoSpin® Gel and PCR Clean-up kit (Machery-Nagel, Düren, Germany) was used for PCR clean-up and plasmids were isolated using the GeneJET Plasmid Miniprep Kit (Thermo Fisher Scientific, Waltham, USA). Plasmid pNW33Nkan backbone was cut with restriction enzyme *BamH*I (Thermo Fisher Scientific, Waltham, USA) and inserts were amplified using Allin HiFi DNA polymerase (HighQu, Kraichtal, Germany) and the overlapping regions joined by Gibson assembly [39]. Taq polymerase (New England Biolabs) was used as mentioned above for colony PCR and primer characteristics and sequences are isted in Additional file 1: Table S1. The correctness of inserted DNA sequences was confirmed by sequencing. The constructed plasmids were named pP2pyk-*gfpUV* or pP2pyk_TPP-*gfpUV* and transformed to *P. riograndensis* SBR5 via magnesium-aminoclay method as described by Brito et al. (2016) [22].

### Fluorescence-activated cell scanning analysis

To quantify the fluorescence intensities, SBR5 cells were analyzed by using flow cytometry. Routinely, the SBR5 transformants were grown until reaching the middle of the exponential growth phase and centrifuged for 15 min at 4000 rpm. The pellets were washed 3 times in NaCl 0.9% solution and the OD_600nm_ was adjusted to 0.3. The fluorescence of the cell suspension was measured by using flow cytometer (Beckman Coulter, Brea, US) and the data analyzed in the Beckman Coulter Kaluza Flow Analysis Software. The settings for the emission signal and filters within the flow cytometer for detection of GfpUV were 550 short pass and 525 band pass in FL9 filter. In order to compare the obtained values of median fluorescence intensity (MFI), the results were tested for significance using one-way ANOVA followed by post hoc comparisons using the Tukey’s honest significant difference (HSD) test. The level of significance of the differences observed in each strain between the control (0 μM of thiamine) and test conditions (5, 10, 15, 20 and 25 μM of thiamine) was expressed as one star for *p ≤ 0.05. Nonsignificant differences, when *p* > 0.05, were not pointed.

## Results

### Cultivation of *P. riograndensis* SBR5 under various growth conditions

Apart from a core subset of constitutively expressed genes, most genes are transcribed only under certain conditions. In order to obtain a broad representation of the whole transcriptome, we performed several shaking flasks cultivations of SBR5 under different conditions for subsequent RNA extraction. In standard conditions, such as growth in PbMM medium with pH 7 and growth in LB medium at 30 °C, the biomass (ΔOD) was approximately 1.35 and the growth rate (μ) approximately 0.5 h^-1^ (Additional file 2: Table S2). Compared to standard conditions, growth of SBR5 under stress conditions was in general slower (Additional file 2: Table S2). Hyperosmotic stress, low or high pH and low temperature (20 °C) affected growth of SBR5 to the largest extent (Additional file 2: Table S2). Under all conditions, exponentially growing cells were harvested for RNA isolation.

### RNA-seq experiment of *P. riograndensis* SBR5

After confirmation of RNA integrity and absence of DNA contamination, the prepared RNA samples were pooled. The total number of reads generated from whole transcriptome and 5′-end enriched libraries were 11.57 million and 1.40 million, respectively (Table 2). Trimming of the reads with a length threshold of 20 base pairs resulted in 5.87 million (51% of the total reads) remaining reads for the whole transcriptome library and 827,376 (59% of total reads) for the 5′-end enriched library (Table 2). The trimmed reads were mapped to the genome of *P. riograndensis* SBR5, and 1.22 million whole transcriptome library reads and 345,313 reads of the 5′-end enriched library were uniquely aligned to the genome of SBR5 while 122,980 and 31,899 reads were aligned to multiple genome regions, respectively (Table 2).

**Table 2:** Sequencing and mapping features of cDNA libraries of *P. riograndensis* SBR5

	Whole transcriptome	5’ enriched ends
Total reads	11,577,588	1,401,776
Reads after trimming	5,876,240	827,376
Mapped reads	1,351,334	345,313
Mapped at single position	1,228,354	313,414
Mapped at multiple position	122,980	31,899

### Identification of transcription start sites (TSS) based on the mapped 5′-end enriched data

**Fig. 1:**
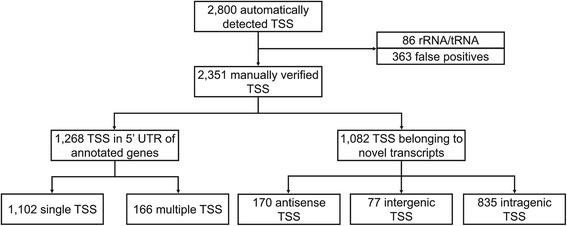
Classification of TSS identified with RNA-seq. Schematic view of the TSS analysis flow: TSS automatic identification by ReadXplorer [25], filtering of false positives and rRNA/tRNA, manual verification and classification of TSS between TSS belonging to 5′ UTR of annotated genes or to novel transcripts

In order to detect putative TSS in the mapped 5′-end enriched data; 2 TSS analysis parameter sets were chosen (Table 1). The use of the parameter set 1 led to the automatic detection of 849 TSS and 1951 TSS were detected by using parameter set 2 (Table 1). Subsequently, these results were merged. Figure 1 shows the scheme of the manual review of the automatically detected TSS which led to the identification of 86 TSS belonging to rRNA or tRNA genes. Moreover, 363 elements were considered not to be TSS or to be false positives. TSS were considered false-positives if no clear accumulation of read starts was observed at the particular genomic position and additionally the putative TSS was detected within an uneven gradient of accumulated read starts [16]. The 2351 remaining TSS were classified as either belonging to 5′ UTRs of annotated genes or of novel transcripts. Out of the 6705 genes annotated in the genome of SBR5 [12], 1173 were found to possess TSS. The detected TSS were classified as single when only one TSS was present upstream a gene (1102) or multiple when more than one TSS were present upstream a gene (166). The remaining 1082 TSS were classified as belonging to novel transcripts, divided into the groups of antisense when the transcript was located in the antisense orientation to an annotated gene (170), intergenic when the transcript was located between annotated genes (77) or intragenic when a TSS was located within annotated genes in sense orientation (835) (Fig. 1).

### Distribution of 5′ UTR length in *P. riograndensis* SBR5

**Fig. 2:**
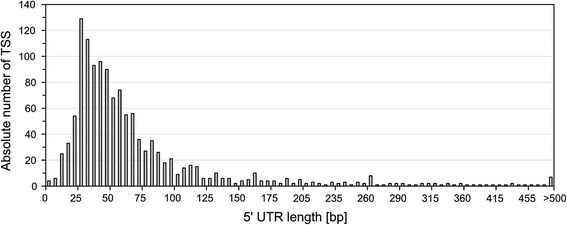
Distribution of 5′ UTR lengths of mRNAs assigned to genes in *P. riograndensis* SBR5. The 5′ UTR length was the distance between the identified TSS and its assigned TLS. The lengths of the 1269 5′ UTRs of annotated genes were grouped in a crescent interval of 5 base pairs or longer than 500 base pairs

The sequences located between TSS and the gene start codons were used for the analysis of 5′ UTR lengths. For this purpose, only the 5′ UTRs assigned to annotated genes were considered. The length of 5′ UTRs in *P. riograndensis* varied from 0 to 799 base pairs. Only 2 of the genes with annotated TSS were considered leaderless (no 5′ UTR present): P.riograndensis_final_2873 coding for stress-induced protein and P.riograndensis_final_5691 coding for hypothetical protein (Additional file 3: Table S3). Moreover, 10 of the analyzed 5′ UTRs were found to be shorter than 10 base pairs (Additional file 3: Table S3). Figure 2 shows the distribution of the 5′ UTR lengths indicating that the majority of 5′ UTRs are 25 to 50 base pairs long. Among the 1269 analyzed 5′ UTRs, 209 (16.4%) were longer than 100 base pairs (Fig. 2). Those 5′ UTRs were further used in a screen for *cis*-regulatory RNA elements.

### Identification of consensus promoter motif sequences in *P. riograndensis* SBR5

**Fig. 3:**
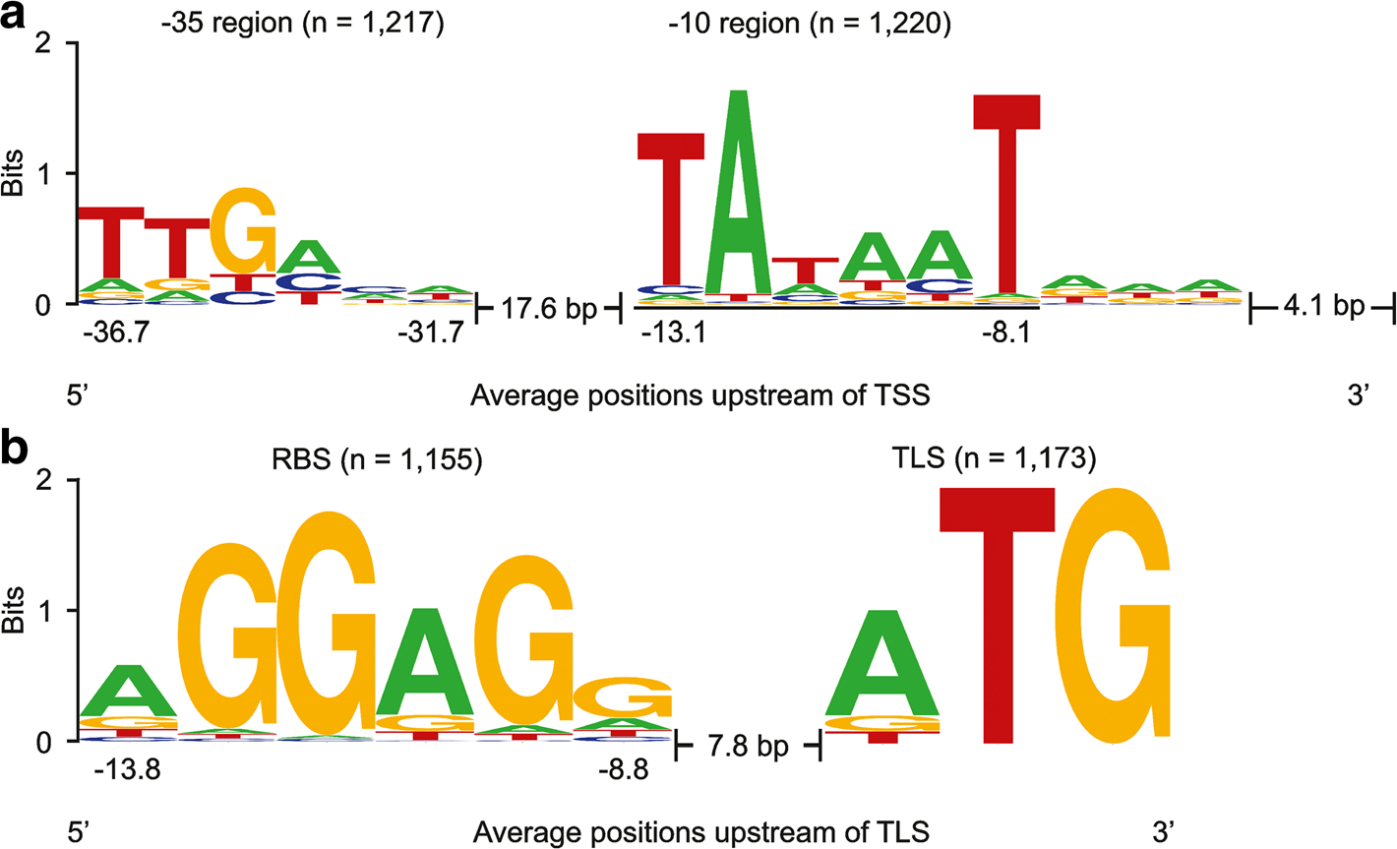
Analysis of promoter, ribosome binding site and translation start site motives in *P. riograndensis* SBR5. The nucleotide distribution in the promoter motifs (**a**), ribosome binding sites and translation start sites (**b**) of *P. riograndensis* SBR5 were determined by using the Improbizer tool [32]. WebLogo tool [33] was used to determine the conservation of the nucleotides which was measured in bits and represented in the plot by the size of the nucleotide

The 1269 TSS identified as belonging to annotated genes were used in a search for the conserved promoter motifs (Fig. 1). The software Improbizer was applied to predict the motifs in a DNA region 70 base pairs upstream of each of those TSS [32]. Conserved −35 and −10 promoter sequence motifs were found in 1220 (96.1%) and 1217 (95.9%) of the analyzed sequences, respectively (Fig. 3A). Figure 3A shows the −10 and −35 motif sequence logos, which were ttgaca for −35 hexamer motif and TAtaaT for the −10 hexamer motif. The mean spacer lengths between the −35 and −10 motifs and −10 motifs and TSS were 17.6 base pairs and 4.1 base pairs, respectively (Fig. 3A).

### Identification of RBS (ribosome binding site) and TLS (translation start site) consensus sequences in *P. riograndensis* SBR5

Similarly to the analysis of the promoter motifs, the Improbizer software [32] was used to determine the consensus sequence of RBS and TLS in the sequence 50 base pairs upstream of the translation start codon of genes associated to the 1269 previously identified TSS (Fig. 1). Some genes were characterized as associated to multiple TSS (Fig. 1), therefore the upstream sequence of these genes was only included once in the analysis. Hence, the 1173 remaining sequences were extracted from the genome of SBR5 and submitted to Improbizer [32] and WebLogo [33] for the identification of the conserved motifs of RBS and TLS (Fig. 3B). RBS motifs were identified in 98% (1155) of analyzed sequences. The determined RBS motif aGGaGg of *P. riograndensis* SBR5 includes 3 conserved guanines in approximately 90% of the analyzed sequences (Fig. 3B). Translational start codons were identified in all the analyzed sequences (Fig. 3B). The TLS found in the analyzed sequences were ATG (924; 79%), GTG (138; 12%) and TTG (111; 9%). The lengths of the spacer sequence between RBS and TLS varies between 5 and 13 base pairs, with an average of 7.8 ± 2.0 base pairs (Fig. 3B).

### Identification of cis-regulatory elements in 5′ UTRs of *P. riograndensis* SBR5 genes

In order to identify putative RNA motifs in the genome sequence of *P. riograndensis* SBR5, we used the Infernal tool [26] and the Rfam database, which contains hundreds of RNA families [27]. This approach revealed 327 RNA motifs that subsequently were manually cross checked. Matches to tRNAs, ribosomal RNAs and RNA motifs from Eukaryotes or different bacterial groups were not considered. As result, 98 RNA motifs among 31 Rfam families were identified (Additional file 4: Table S4).

In an alternative approach based on the RNA-seq data, we analyzed 209 5′ UTRs longer than 100 base pairs (Fig. 2) for the presence of *cis*-regulatory elements by comparison to the Rfam database. This analysis revealed the presence of 11 putative *cis*-regulatory elements grouped in 9 types of riboswitch families (Table 3). Thus, based on the RNA-seq data, the existence of 11 out of 98 putative 5′ UTR RNA motifs upstream of annotated genes was confirmed. A TPP (thiamine pyrophosphate) sensitive riboswitch was predicted to be present in the 5′ UTR of the gene P.riograndensis_final_150 (*thiC*) encoding phosphomethylpyrimidine synthase, which is putatively involved in thiamine biosynthesis, and in the 5′ UTR belonging to the operon P.riograndensis_final_504–502. Although P.riograndensis_final_503 gene is automatically annotated as a hypothetical protein, BLASTx analysis revealed that it belongs to the thiamine-biding protein superfamily. More vitamin and amino acid related riboswitches were found: a pantothenate related *pam* riboswitch in the 5′ UTR of putative pantothenate synthesis operon and a riboswitch recognizing S-adenosylmethionine (SAM) in the 5′ UTR of an operon encoding homoserine O-succinyltransferase and cystathionine gamma-lyase proteins. The T-box regulatory elements were found in 5′ UTR of the genes coding for D-3-phosphoglycerate dehydrogenase (*serA*) and valine tRNA ligase (*valS*). Furthermore, the protein dependent L20 leader and L21 leader riboswitches, the metabolite dependent *ydaO-yuaA* riboswitch, the *pfl* riboswitch and the glycine dependent riboswitch were identified in this work (Table 3).

**Table 3:** Riboswitches detected in the transcriptome of *P. riograndensis* SBR5 and their transcriptional organization

No.	Accession	Riboswitch and its transcriptional organization	Related function	Locus tag
1	RF00059	(TPP)-*thiC*	Phosphomethylpyrimidine synthase	P.riograndensis_final_150
2	RF00059	(TPP)-P.riograndensis_final_504-P.riograndensis_final_503-P.riograndensis_final_502	Conserved hypothetical protein- Hypothetical protein- Biding protein dependent transport system inner membrane component	P.riograndensis_final_504-P.riograndensis_final_503-P.riograndensis_final_502
3	RF00162	(SAM)-*metA*- P.riograndensis_final_2059	Homoserine O-succinyltransferase-Cystathionine gamma-lyase	P.riograndensis_final_2058-P.riograndensis_final_2059
4	RF00230	(T-box)-*serA*	D-3-phosphoglycerate dehydrogenase	P.riograndensis_final_4453
5	RF00230	(T-box)-*valS*	Valine tRNA ligase	P.riograndensis_final_5318
6	RF00379	(ydaO-yuaA)-P.riograndensis_final_93	Cell wall-associated hydrolase (invasion- associated protein)	P.riograndensis_final_93
7	RF00504	P.riograndensis_final_6104-*gcvPA*-*gcvPB*	Glycine utilization	P.riograndensis_final_6104-P.riograndensis_final_6105-P.riograndensis_final_6106
8	RF00558	(L20 leader)-*infC*- P.riograndensis_final_1528-P.riograndensis_final_1529	Translation initiation factor IF-3- Conserved hypothetical protein- Ribosomal protein L20	P.riograndensis_final_1527-P.riograndensis_final_1528-P.riograndensis_final_1529
9	RF00559	(L21 leader)-*rplU*- P.riograndensis_final_5299-P.riograndensis_final_5300	50S ribosomal protein L21- Conserved hypothetical protein- 50S ribosomal protein L27	P.riograndensis_final_5298-P.riograndensis_final_5299-P.riograndensis_final_5300
10	RF01749	(pan)-*panB- panC-* P.riograndensis_final_4379	3-Methyl-2-oxobutanoatehydroxymethyltransferase-Pantothenate synthetase- Aspartate 1-decarboxylase alpha	P.riograndensis_final_4381-P.riograndensis_final_4380-P.riograndensis_final_4379
11	RF01750	(pfl)-P.riograndensis_final_6217	Hypothetical protein	P.riograndensis_final_6217

### A TPP riboswitch influences *gfpUV* expression in *P. riograndensis* SBR5

**Fig. 4:**
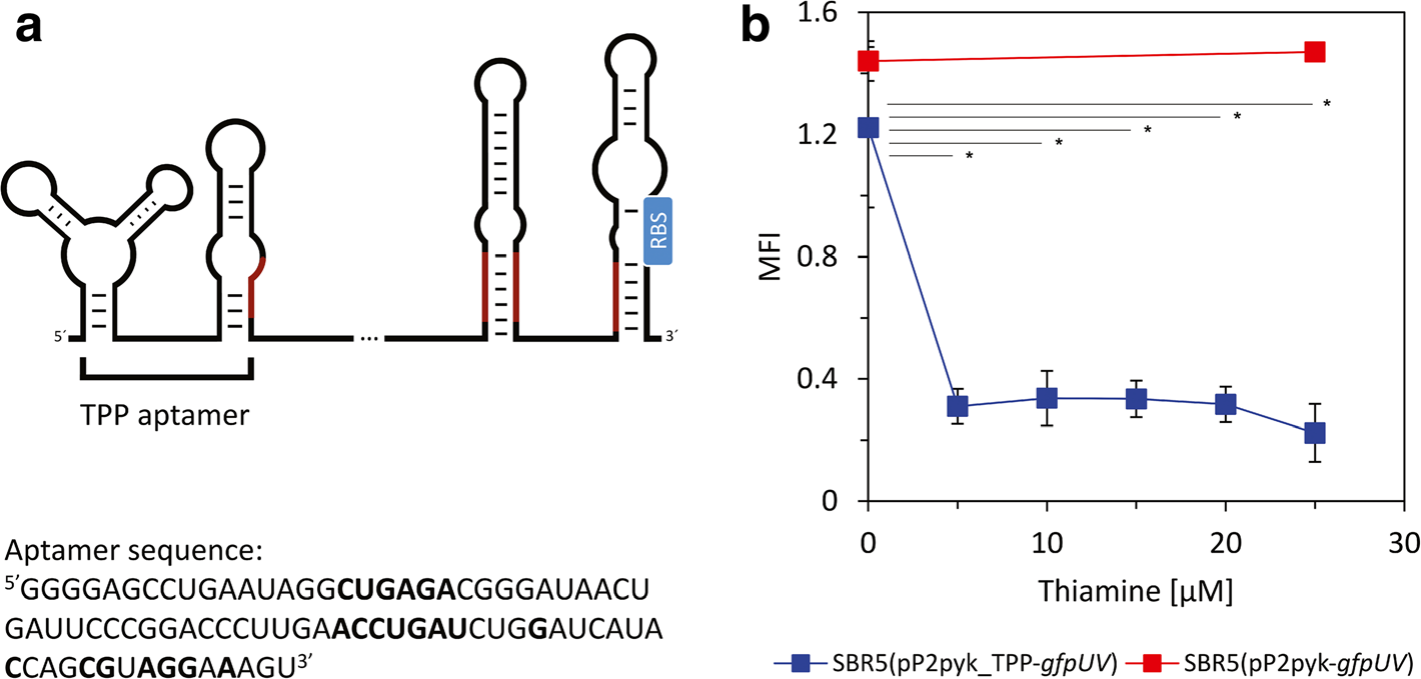
TPP riboswitch influence on the reporter *gfpUV* gene expression of *P. riograndensis* SBR5. **a**. Schematic representation of the TPP riboswitch and TPP aptamer sequence predicted using RNAfold tool [31]; regions of riboswitch scheme in red represents possible anti-sequestering stems present in the riboswitch sequence; regions of aptamer sequence in bold are identical to the TPP riboswitch consensus sequence of *B. subtilis* **b**. GfpUV median fluorescence intensity (MFI) in SBR5 under 6 gradually increasing concentrations of thiamine; *gfpUV* expression was driven either by the *pyk* promoter with 5′ UTR exchanged by the *thiC* gene 5′ UTR or *pyk* promoter carrying native 5′ UTR. Means and standard deviation of biological triplicates were measured by flow cytometry of 20,000 cells. Under one-way between subjects ANOVA followed by post hoc comparisons using the Tukey’s HSD test, the level of significance of the differences observed in each strain between the control (0 μM of thiamine) and test conditions (5, 10, 15, 20 and 25 μM of thiamine) is represented as one star (*p ≤ 0.05). Nonsignificant differences, when *p* > 0.05, are not pointed

Riboswitches are regulatory elements found in the 5′-UTR of genes and they perform the regulatory control over the gene transcript by directly binding a small ligand molecule. In the riboswitch sequence, an aptamer domain recognizes and binds to that ligand which leads to adopting a new conformation that interfaces with the gene transcriptional (presence of terminator sequence) or translational machinery (sequestration of the RBS by a stem) [40]. The prediction of the secondary structure of the TPP riboswitch in the 5′ UTR of *thiC* gene, with length of 313 base pairs, showed that it contains no terminator sequence. However, a 5′-GAUAA-3′ sequence and its complementary 5′-UUAUC-3′ is present in many of the predicted stems, including the stems of the aptamer region (Fig. 4A). This indicates the existence of anti-sequestering stems in this molecule, as showed schematically in Fig. 4A. Furthermore, we aimed to detect the influence of the *P. riograndensis* TPP riboswitch on gene expression in the presence of different concentrations of its ligand thiamine by measuring the GfpUV fluorescence. SBR5 cells were transformed with the plasmid pP2pyk_TPP-*gfpUV* which carries the constitutive promoter Ppyk with its native 5′ UTR replaced by the 5′ UTR of the P. riograndensis_final_150. The so changed Ppyk promoter drives the expression of the reporter gene *gfpUV* (Additional file 1: Table S1). As shown before, the 5′ UTR of the P. riograndensis_final_150 gene contains the sequence of a TPP riboswitch (Table 3). As control for this assay, the plasmid pP2pyk-*gfpUV*, containing Ppyk native 5′ UTR was used to transform SBR5 cells and the resultant strain was also cultivated in glucose PbMM, but supplied with 0 or 25 μM of thiamine. The MFI of the control strain SBR5(pP2pyk-*gfpUV*) remained the same when the cells were in absence or in presence of 25 μM of thiamine (Fig. 4B). The GfpUV MFI of SBR5(pP2pyk_TPP-*gfpUV*) was similar to the control strain when no thiamine was added to the growth medium (Fig. 4B). In contrast, there was a significant effect of thiamine on MFI of SBR5(pP2pyk_TPP-*gfpUV*) at the *p* < 0.05 level for the tested conditions [F (5, 12) = 17.8, *p* = 0.00004]: post hoc comparisons using the Tukey’s HSD test indicated that the mean score for the 0 μM thiamine was significantly different than the 5, 10, 15, 20 and 25 μM thiamine conditions. However, the MFI of SBR5(pP2pyk_TPP-*gfpUV*) in 5, 10, 15, 20 and 25 μM thiamine conditions did not significantly differ from one another (Fig. 4B).

### Identification and characterization of novel transcripts

Here, we performed the characterization of *P. riograndensis* novel transcripts based on the 5′-end enriched data set. Among the 2351 manually verified TSS, 1082 were classified as belonging to novel transcripts. Depending on their position in genes or untranslated regions, these TSS belonged to antisense transcripts (170), transcripts intragenic (835) to annotated genes or their 5′/3′ UTRs, or intergenic (77) transcripts (Fig. 1). Additional file 5: Table S5 shows the intragenic transcripts which were organized according to their position and associated gene. As intergenic novel transcripts could not be assigned to annotated genes, they were manually annotated as unknown transcripts (Additional file 6: Table S6). BLAST analysis of the intergenic novel transcripts resulted in discovery of 34 small proteins and 27 small RNAs. Small RNAs were analyzed in the Rfam database and 3 of them were annotated as Small SRP (P.riograndensis_final_s0002), BsrC sRNA (P.riograndensis_final_s0008) and RNase P (P.riograndensis_final_s0013) (Table 4). All others were assigned an unknown function.

**Table 4:** Novel transcripts with known function in *P. riograndensis* SBR5

Feature	Class	Locus tag	Feature start	Feature stop	Length	Strand
Small SRP	Small RNA	P.riograndensis_final_s0002	130,367	130,639	272	+
BsrC sRNA	Small RNA	P.riograndensis_final_s0008	688,067	687,745	322	–
RNase P	Small protein	P.riograndensis_final_s0039	6,002,090	6,001,625	465	–

### Gene expression ranked according to transcript abundances

The abundance of transcripts in the analyzed RNA samples was quantified on the basis of the whole transcriptome dataset using RPKM values. Transcripts were detected in 6367 of the coding sequences during the analysis, corresponding to 94% of the total number of genes annotated in the genome of *P. riograndensis*. Transcript abundance varied over 6 orders of magnitude with RPKM values ranging from 0.11 to 71,849.57 and was categorized arbitrarily as follows. Transcript abundance was considered low for approximately 70% of transcripts (with RPKM values <100), intermediate (RPKM between 100 and 1000) for around 25% of the detected transcripts and high for approximately 5% of the transcripts (RPKM between 1000 and 10,000). Twenty one transcripts showed RPKM values exceeding 10,000 and these were considered as transcripts with very high transcript abundance and are listed in Table 5.

**Table 5:** Most abundant transcripts of *P. riograndensis* SBR5 under the chosen cultivation conditions

Gene	Product	RPKM Value
*rpsH*	30S ribosomal protein S8	71,849.57
P.riograndensis_final_4321	N-acetyltransferase superfamily	70,789.99
P.riograndensis_final_30	Veg protein; sporulation, Stimulates biofilm formation via transcriptional activation of extracellular matrix genes	53,361.67
P.riograndensis_final_5486	Hypothetical protein	39,913.22
P.riograndensis_final_2764	Hypothetical membrane protein	28,462.66
P.riograndensis_final_2316	Small, acid-soluble spore protein superfamily	24,204.00
P.riograndensis_final_1999	PTS maltose transporter subunit IIBC	21,134.31
P.riograndensis_final_6014	50S ribosomal protein L24	20,187.98
P.riograndensis_final_4594	Hypothetical protein	18,591.09
P.riograndensis_final_2529	Hypothetical protein	17,946.18
P.riograndensis_final_956	Recombinase RecA	17,771.90
P.riograndensis_final_5132	Ribosomal S21 superfamily	17,463.97
P.riograndensis_final_5601	Small, acid-soluble spore protein superfamily	16,757.65
P.riograndensis_final_1944	Protein of unknown function DUF1292 superfamily	15,456.74
ftsH	ATP-dependent zinc metalloprotease FtsH	15,355.15
rpsS	30S ribosomal protein S19	15,060.69
P.riograndensis_final_6183	Conserved hypothetical protein	14,247.19
P.riograndensis_final_6034	50S ribosomal protein L7A	14,218.27
P.riograndensis_final_1943	Crossover junction endodeoxyribonuclease RuvA	11,659.67
P.riograndensis_final_1181	Transcriptional regulator, TetR family	11,466.86
P.riograndensis_final_6018	50S ribosomal protein L16	10,826.18

Gene products in italics were predicted with BLASTx analysis

BLASTx analysis was performed to predict the functions (conserved protein domains) of the 14 genes which were automatically annotated as hypothetical proteins or as proteins with unknown function. However, a function could not be predicted for 5 genes with very highly abundant transcripts (Table 5). Part of the very highly abundant transcripts code for ribosomal proteins (6 genes). Remarkably, 3 genes related to bacterial sporulation had very highly abundant transcripts (Table 5).

### Identification of operon structures in *P. riograndensis* SBR5

**Fig. 5:**
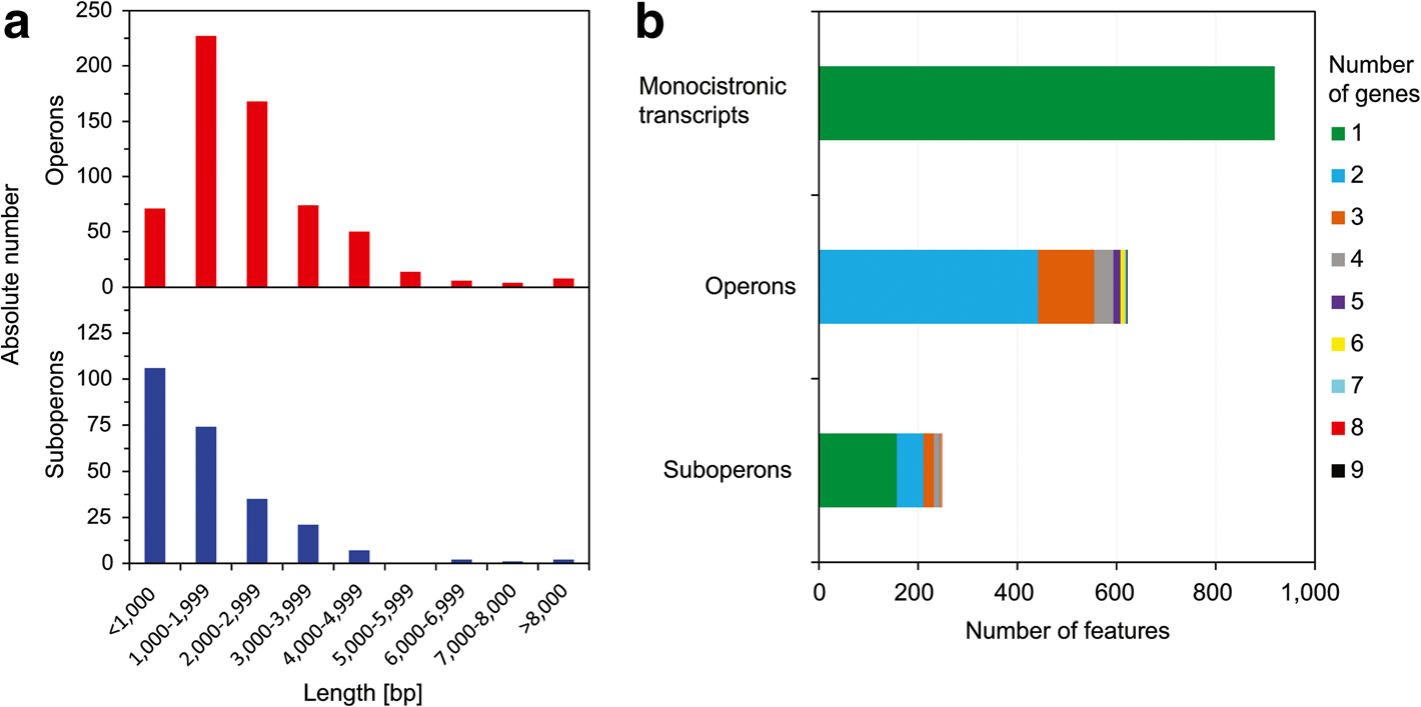
Operon analysis in *P. riograndensis* SBR5. **a.** Length distribution (in base pairs) of detected operons and suboperons; **b**. Analysis of feature number in monocistronic transcripts, operons and suboperons in *P. riograndensis* SBR5

Based on the mapped reads generated from whole transcriptome library, we assigned genes either to monocistronic transcripts, primary operons or suboperons. Genes were assigned to suboperons when a TSS was detected internal to the operon sequence. Genes with annotated TSS that were not automatically detected as primary operons were classified as monocistronic transcripts. In total, 919 monocistronic transcripts were detected, and 1776 genes were assigned to 622 operons and 248 suboperons (Fig. 5B). The length distribution of the operons and suboperons was estimated and shown to peak between 1000 and 3000 base pairs for operons, while the majority of the suboperons were shorter than 2000 base pairs (Fig. 5A). In general, the number of operons decreases with the increasing number of genes in those operons and most operon structures (71%) are composed of 2 genes while only 5 operons contained more than 7 genes (Fig. 5B). Two operon structures which are putatively involved in Fe^3+^ siderophore uptake and transport could be detected: *fhuB* - P. riograndensis_final_3660 (Fe^3+^ hydroxamate import system permease and component of an ABC type Fe^3+^ siderophore transport system, respectively) and P. riograndensis_final_5688 - P. riograndensis_final_5687 which encodes a Fe^3+^ siderophore ABC transporter permease (Additional file 3: Table S3). However, known operons comprising nitrogen fixation genes [10] were not detected in the present study. Notably, riboswitches were found in the 5′ UTRs of 6 operons P.riograndensis_final_502–504, *infC*-P.riograndensis_final_1528–1529, *metA*-P.riograndensis_final_2059, *panB-panC-*P.riograndensis_final_4379, *rplU*-P.riograndensis_final_5299–5300 and P.riograndensis_final_6104-*gcvPA*-*gcvPB* (Table 3).

## Discussion

In the present study, we performed for the first time a detailed transcriptome analysis of *P. riograndensis* SBR5. This work lays a foundation for understanding of gene expression in this bacterium and complements differential gene expression analysis. To enable the comprehensive characterization of ‘static’ bacterial transcriptomes it is necessary to generate a pool of different transcripts in order to obtain the expression of as many genes as possible [16]. This was largely achieved by cultivation of *P. riograndensis* SBR5 under 15 distinct conditions and pooling RNA samples prior to sequencing, since we found 94% genes expressed under these conditions. However, the absolute number of TSS (present in 5′ UTR of annotated genes together with TSS belonging to novel transcripts) in *P. riograndensis* SBR5 was comparable to that in *B. methanolicus* MGA3 [16] and *C. glutamicum* [15] (equaled 2350, 2167, and 2591, respectively) although the genome of *P. riograndensis* SBR5 is more than 2 fold larger than those of *B. methanolicus* MGA3 [41] and *C. glutamicum* [42]. This observation may also reflect the fact that RNA was pooled from cells cultivated under various growth and stress conditions that were chosen with a similar rationale for the 3 bacteria and may indicate that a similar set of genes is transcribed under the chosen conditions.

In this study, the 5′ UTR length of *P. riograndensis* SBR5 transcripts was shown to be equal or longer than 10 nucleotides in 99.2% of the cases and it peaked at around 30 base pairs (Fig. 2). A similar 5′ UTR length distribution can be found in *Actinoplanes sp., C. glutamicum* and *B. methanolicus* [15, 16, 43]. However, leaderless transcripts are rare in *P. riograndensis* SBR5 as the present study only revealed 2 transcripts (P.riograndensis_final_2873 and P.riograndensis_final_5691) to be leaderless in this firmicute (Additional file 3: Table S3). In silico analysis performed by Zheng et al. (2011) [44] showed that 207 among 953 analyzed bacterial genomes possess leaderless genes including species of the *Firmicutes* and *Actinobacteria* phyla. The scarcity of leaderless transcripts in the transcriptomes of the low-GC Gram-positives *B. methanolicus* [16] and *P. riograndensis* contrasts with the large proportion of leaderless transcripts present in the high-GC Gram-positive actinobacteria *Actinoplanes sp.* (20%) [43] and *C. glutamicum* (33%) [15].

The analysis of the promoter, RBS and TLS motives in *P. riograndensis* SBR5 transcriptome revealed that the RBS consensus sequence aGGaGg, TLS frequency (ATG represented 79% of the analyzed sequences and GTG and TTG represented 12% and 9%, respectively) and spacing between RBS and TLS (7.8 base pairs) in *P. riograndensis* SBR5 corresponds well to the conserved sequences motifs historically found in bacteria [45–47]. While the −10 region (TAtaaT), spacing between −10 and −35 boxes (17.6 base pairs) and between −10 box and TSS (4.1 base pairs) are conserved between *P. riograndensis* SBR5, *E. coli, B. subtilis* and *B. methanolicus* [16, 48, 49], the conserved −35 region (ttgaca) in SBR5 was similar only to the −35 box described for other bacilli [16, 50].

Riboswitch-mediated control of expression of a variety of genes in bacteria could have practical implications, such as development of new antibacterial drugs [21], or more generally contribute to improvement of the understanding of bacterial metabolism. Here, a genome-based riboswitch analysis revealed 98 putative RNA motifs, 11 of which were also detected in the sequenced RNAs (Additional file 4: Table S4). In *Firmicutes*, the SAM riboswitch is part of the S-box group of riboswitches which are involved in regulation of SAM, cysteine and methionine biosynthesis, and sulfur metabolism [51, 52]. This type of riboswitch has been well characterized in bacilli, for example in *B. subtilis*, which has at least 11 operons and 26 genes under control of S-box RNA [53]. S-box RNA from *B. subtilis* directly senses the level of SAM and functions as SAM dependent riboswitch [54]. However, the most frequent mechanism of riboswitch regulation of amino acid operons in the *Firmicutes* is the T-box regulatory system [55, 56]. In *B. subtilis* and other *Firmicutes*, the T-box can regulate many genes encoding amino acid biosynthetic enzymes and transporters [57]. The *ydaO-yuaA* riboswitches occur upstream of these 2 genes in *B. subtilis* and operate as a genetic “off” switch [58]. Furthermore, recognition of the cyclic di-AMP by *ydaO-yuaA* was characterized and also shown to exist in *B. subtilis* [59, 60]. The riboswitches in *P. riograndensis* SBR5 identified in the present study need to be investigated to some detail to unravel their regulatory function.

In order to analyze the function of 1 exemplary riboswitch found in the transcriptome of *P. riograndensis* SBR5, we selected the TPP dependent riboswitch present upstream of the *thiC* gene (Table 3) to control the expression of *gfpUV*. The gene *thiC* encodes a phosphomethylpyrimidine synthase involved in TPP biosynthesis [61]. The *E. coli thiC* riboswitch controls translation initiation and, in the presence of TPP *thiC* product is not translated [62, 63]. The thiamine analog triazolethiamine showed a concentration dependent reporter gene repression by the TPP riboswitch in the 5′ UTR of the thiamine kinase *thiK* [62]. In the present study, thiamine was added to the growth medium and already 5 μM thiamine fully reduced *gfpUV* expression (Fig. 4B). *P. riograndensis* SBR5 is a thiamine prototroph capable of growing in minimal medium without added thiamine (data not shown). The *gfpUV* expression without additional thiamine remained high (Fig. 4B) suggesting that the amount of thiamine synthetized by SBR5 did not activate the TPP riboswitch. Riboswitch aptamers remain highly conserved through evolution because each one must preserve selective binding of its target metabolite. Hence, the conserved TPP riboswitch consensus regions of *B. subtilis* were also present in the TPP riboswitch aptamer sequence targeted in this study (Fig. 4A) [21]. The secondary structure analysis showed that, in contrast to the *tenA* TPP riboswitch in *B. subtilis* [21], the SBR5 *thiC* TPP riboswitch does not possess a transcriptional terminator sequence. This led us to investigate the presence of sequestering/anti-sequestering stems that could participate in the “on/off” state of the *P. riograndensis* SBR5 *thiC* TPP riboswitch. The schematic representation of the *thiC* TPP riboswitch secondary structure shows that the RBS is sequestered within a stem-loop structure predicted to inhibit translation initiation (Fig. 4A). We could detect the sequence 5′-GATAA-3′ and its complementary region 5′-UUAUC-3′ inserted in the TPP aptamer sequence, in the stem-loop containing the *thiC* RBS and also in the sequence of 1 stem-loop located before RBS containing stem. The locations of these sequences inside the TPP riboswitch structure are decipted in red in the schematic representation of Fig. 4A. Comparable secondary structures and gene expression control have very recently been described for the *E. coli thiC* TPP riboswitch [63]. *P. riograndensis* possesses 3 further putative TPP riboswitches, one of which was expressed under the growth conditions of the RNA-seq analysis presented here (Table 3; Additional file 4: Table S4). Although they share conserved sequences and secondary structure predictions (data not shown) it remains to be studied if these putative TPP riboswitches are indeed responsive to TPP and if they operate as transcriptional or translational riboswitches.

Landscape transcriptome analyses are suitable to identify antisense, intragenic or intergenic novel transcripts [15, 16]. Sixteen percent of the 1082 novel transcripts identified in the transcriptome of *P. riograndensis* SBR5 were shown to be antisense (Fig. 1). The number of reported antisense RNAs varies between bacteria and the biological advantages of such overlapping transcription remains unclear, but antisense RNAs may play important roles in regulation e.g. by transcription interference [64]. Commensurate with this notion, we could identify that transcription of 3 antisense RNAs initiates in the 5′ UTRs of the genes on the complementary strand and, thus, antisense transcription may interfere or attenuate with their transcription (P.riograndensis_final_5580, P.riograndensis_final_6016 and P.riograndensis_final_6182; Additional file 5: Table S5). Moreover, 77 novel transcripts were identified in the intergenic regions between previously known genes, part of the identified intergenic transcripts represent small RNA and small protein genes (Additional file 7: Table S7). Of these, 34 were predicted to encode small proteins, for example, small signal recognition particle (or small SRP; P.riograndensis_final_s0002), which is known to be involved in protein targeting in other bacteria [65]. Twenty seven small RNA genes were found, e.g. the small RNA *bsrC* (P.riograndensis_final_s0008), which is present also in *B. subtilis* [66] and RNase P (P.riograndensis_final_s0013), the ubiquitous endonuclease that catalyzes the maturation of the 5′ end of the tRNAs [48]. Overall, the function of the novel intergenic transcripts representing small protein and small RNA genes still has to be elucidated.

In the transcriptome of *P. riograndensis* SBR5, the abundantly transcribed genes could be grouped by their presumed functions: ribosomal proteins, sporulation related proteins, proteins related to carbon metabolism and others. Many abundantly transcribed genes encode proteins of unknown function (Table 5). Among the highly expressed genes, we have detected 1 gene coding for a subunit of a maltose phosphotransferase system (P.riograndensis_final_1999, Table 5). Maltose is a disaccharide formed from two units of glucose and this organic compound can be identified in root exudates of different plant species [65]. Although maltose was not utilized as carbon source in the present study, the transcription of this carbohydrate phosphotransferase system gene may be due to the fact that we mostly used glucose as carbon source. The gene encoding a glucose specific phosphotransferase system (P.riograndensis_final_1998) had RPKM value of 156.17 (data accession GSE98766) which places it in the group of intermediately abundant transcripts. Thus, the affinity of the highly transcribed maltose transporter to glucose still remains to be studied. A transcriptome analysis of carbon source utilization (β-glucan, starch, cellobiose, maltose, glucose, xylose and arabinose) by *Paenibacillus* sp. JDR-2 revealed a regulatory connection for the utilization of the polysaccharides β-glucan, starch and xylans, while transcription of genes coding for proteins involved in monosaccharide (e.g. arabinose and glucose) utilization was less apparent [13]. BLASTx analysis revealed 3 sporulation-related genes (P.riograndensis_final_4321, P.riograndensis_final_2316 and P.riograndensis_final_5601) among the most abundantly expressed genes (Table 5). This result might be due to the fact that different stress conditions were applied during cultivations of SBR5, which included 5 min of cold and heat shock but also exposure to salinity, solvent, low temperature and low pH along the bacterial growth. The exposure to stress conditions affected growth rates in comparison to the optimal growth conditions, and might have also induced expression of sporulation related genes (Additional file 3: Table S2). Very recently, sporulation genes *spoVT* and *spoIIIAH* were shown to be transcribed by *P. riograndensis* SBR5 under iron-limiting conditions [67]. Moreover, the related *P. polymyxa* SC2 expressed sporulation genes (*spo0A*, *spoIIE*, *spoIIAA*, *spoIIAB*, *sigE* and *sigF*) when cultivated under sporulation conditions: on LB agar for 24 h at 37 °C, when most of SC2 step into the progress of sporulation [68].

The transcriptional organization of 1776 genes of *P. riograndensis* SBR5 in 622 operons including 248 suboperons and 919 monocistronically transcribed genes (Fig. 5B) was comparable to that found in *B. methanolicus*, in which 1164 genes were assigned to 381 operons and 94 suboperons, and also ~ 900 monocistronic transcripts were detected [16]. Similarly, in *B. subitilis* 736 regulated operons were found [69] and 1013 genes were organized in 616 operons (including 565 suboperons) for the actinobacterium *C. glutamicum* [15]. Most operons detected here were composed of only 2 genes and were between 1000 and 3000 base pairs in length (Fig. 5). Accordingly, most suboperons comprised only 1 gene and were smaller than 2000 base pairs (Fig. 5). The length distribution of suboperons/operons and the number of genes constituting these are commensurate with the average length of the genes in *P. riograndensis* SBR5 genome of 1008 base pairs (data not shown).

Only two differential gene expression analyses on nitrogen fixation [10] and iron metabolism [67] of *P. riograndensis* SBR5 have been published. The nitrogen fixation genes present in 3 genome clusters were characterized by transcript analysis by quantitative real time-qPCR and shown to be transcribed in the operons *nifB1H1D1K1E1N1X1-orf1-hesA-V*, *nifE2N2X2* and *anfHDGK* [10]. In the present study, these operons that are generally transcribed under poor nitrogen supply conditions [14, 70, 71] were not found to be expressed under the chosen growth conditions since all growth conditions were characterized by sufficient nitrogen concentrations (in LB medium or minimal media with 16 mM ammonium sulfate). By contrast, a different gene related to nitrogen fixation encoding putative nitrogenase (flavodoxin; P.riograndensis_final_4327) was found to be expressed. In the second study, 150 genes of *P. riograndensis* SBR5 were shown to be differentially expressed under iron-replete in comparison to iron-limiting conditions [67]. Surprisingly, a high expression level of the Fe^3+^ siderophore transporter gene *fecE* was observed suggesting that *P. riograndensis* SBR5 can uptake Fe^3+^ siderophore from the environment although it is not able to produce those siderophores itself [67]. Here, we could identify 2 operon structures putatively involved in Fe^3+^ siderophore uptake and transport: the operon *fhuB* - *P. riograndensis*_final_3660 which encodes a putative Fe^3+^ hydroxamate import system permease and a component of an ABC type Fe^3+^ siderophore transport system, respectively, and the operon *P. riograndensis*_final_5688 - *P. riograndensis*_final_5687 which comprises a gene encoding the Fe^3+^ siderophore ABC transporter permease (Additional file 3: Table S3).

## Conclusions

The examination of the whole transcriptome of *P. riograndensis*, which was reclassified recently as *P. sonchi* [72], is a valuable contribution to the understanding of biology of this organism. Moreover, our data validated the uncovering of novel transcripts and the presence of hundreds of operons. Although our study has revealed a functional TPP riboswitch in gene regulation of SBR5, further effort is required to fully elucidate the function of this riboswitch. Finally, the data generated in this study should be valuable for future development of genetic tools for this poorly characterized species as much as for the genus *Paenibacillus*. As our RNA-seq analysis provides new insight into the *P. riograndensis* SBR5 transcriptome at the systems level, it will be a valuable basis for further differential RNA-seq analysis exploring agronomical/physiological aspects of this bacterium, e.g. phosphate solubilization.

## Additional files

Additional file 1: Table S1. Bacterial strains, plasmids and oligonucleotides used in this study. *Overlap regions in bold; oligonucleotide sequencess in low caps were used in RNA samples to detect genomic DNA contamination. (XLSX 9 kb)

Additional file 2: Table S2. Delta OD, growth rate and OD of harvested cells of *P. riograndensis* SBR5 when cultivated in *Paenibacillus* minimal medium (PbMM) or lysogeny broth (LB) with a variation of growth parameters. Cells cultivated at 30 °C and transferred to LB medium at 4 °C or 50 °C for 5 min for application of treatment of *cold shock or ^+^heat shock, respectively. (XLSX 14 kb)

Additional file 3: Table S3. List of CDS of *P. riograndensis* SBR5 with corrected translational start sites. (XLSX 288 kb)

Additional file 4: Table S4. Putative RNA motifs present in the genome of *P. riograndensis* SBR5. Highlighted cells are referent to riboswitches detected in the transcriptome analysis. (XLSX 22 kb)

Additional file 5: Table S5. Novel antisense transcripts of *P. riograndensis* SBR5. (XLSX 16 kb)

Additional file 6: Table S6. Novel intragenic transcripts of *P. riograndensis* SBR5. (XLSX 21 kb)

Additional file 7: Table S7. Novel intergenic transcripts of *P. riograndensis* SBR5. (XLSX 12 kb)

## Abbreviations

5′ UTR: 5′ Untranslated region
BLAST: Basic local alignment search tool
CDS: Coding DNA sequences
HSD: Honest significant difference
LB: Lysogeny broth
MES: 2-(N-morpholino)ethanesulfonic acid
MFI: Median fluorescence intensity
MOPS: 3-Morpholinopropane-1-sulfonic acid
OD: Optical density
PbMM: Paenibacillus minimal medium
PGP: Plant growth promoting
RBS: Ribosome binding site
RPKM: Reads per kilobase per million mapped reads
SAM: S-adenosylmethionine
TAPS: 3-[[1,3-Dihydroxy-2-(hydroxymethyl)propan-2-yl]amino]propane-1-sulfonic acid
TE: Trace element
TLS: Translation start sites
TPP: Thiamine pyrophosphate
TSS: Transcription start sites
